# Fluid-induced harm in the hospital: look beyond volume and start considering sodium. From physiology towards recommendations for daily practice in hospitalized adults

**DOI:** 10.1186/s13613-021-00851-3

**Published:** 2021-05-17

**Authors:** Niels Van Regenmortel, Lynn Moers, Thomas Langer, Ella Roelant, Tim De Weerdt, Pietro Caironi, Manu L. N. G. Malbrain, Paul Elbers, Tim Van den Wyngaert, Philippe G. Jorens

**Affiliations:** 1 Department of Intensive Care Medicine, Antwerp University Hospital, Wilrijkstraat 10 Edegem, B-2650 Antwerp, Belgium; 2 Department of Intensive Care Medicine, Ziekenhuis Netwerk Antwerpen Campus Stuivenberg, Lange Beeldekensstraat 267, B-2060 Antwerp, Belgium; 3 Department of Pharmacy, Ziekenhuis Netwerk Antwerpen Campus Stuivenberg, Lange Beeldekensstraat 267, B-2060 Antwerp, Belgium; 4 Department of Medicine and Surgery, University of Milan-Bicocca, Monza, Italy; 5 Department of Anaesthesia and Intensive Care Medicine, Niguarda Ca’ Granda, Milan, Italy; 6 StatUa, Center for Statistics, University of Antwerp, Prinsstraat 13, B-2000 Antwerp, Belgium; 7 Clinical Trial Center (CTC), CRC Antwerp, Antwerp University Hospital, University of Antwerp, Wilrijkstraat 10, B-2650 Edegem, Belgium; 8 Department of Nephrology, Kliniek Sint-Jan, Kruidtuinlaan 32, B-1000 Brussels, Belgium; 9 Department of Anesthesia and Critical Care, AOU S. Luigi Gonzaga, Department of Oncology, University of Turin, Turin, Italy; 10 Faculty of Engineering, Vrije Universiteit Brussel (VUB), Laarbeeklaan 103, 1090 Jette, Belgium; 11 Department of Intensive Care Medicine, Research VUmc Intensive Care (REVIVE), Amsterdam Medical Data Science (AMDS), Amsterdam Cardiovascular Sciences (ACS), Amsterdam Infection and Immunity Institute (AI&II), Amsterdam UMC, Vrije Universiteit, Amsterdam, The Netherlands; 12 Department of Nuclear Medicine, Antwerp University Hospital, Wilrijkstraat 10 Edegem, B-2650 Antwerp, Belgium; 13 Faculty of Medicine and Health Sciences, University of Antwerp, Universiteitsplein 1 Wilrijk, B-2610 Antwerp, Belgium

## Abstract

**Purpose:**

Iatrogenic fluid overload is a potential side effect of intravenous fluid therapy in the hospital. Little attention has been paid to sodium administration as a separate cause of harm. With this narrative review, we aim to substantiate the hypothesis that a considerable amount of fluid-induced harm is caused not only by fluid volume, but also by the sodium that is administered to hospitalized patients.

**Methods:**

We show how a regular dietary sodium intake is easily surpassed by the substantial amounts of sodium that are administered during typical hospital stays. The most significant sodium burdens are caused by isotonic maintenance fluid therapy and by fluid creep, defined as the large volume unintentionally administered to patients in the form of dissolved medication. In a section on physiology, we elaborate on the limited renal handling of an acute sodium load. We demonstrate how the subsequent retention of water is an energy-demanding, catabolic process and how free water is needed to excrete large burdens of sodium. We quantify the effect size of sodium-induced fluid retention and discuss its potential clinical impact. Finally, we propose preventive measures, discuss the benefits and risks of low-sodium maintenance fluid therapy, and explore options for reducing the amount of sodium caused by fluid creep.

**Conclusion:**

The sodium burdens caused by isotonic maintenance fluids and fluid creep are responsible for an additional and avoidable derailment of fluid balance, with presumed clinical consequences. Moreover, the handling of sodium overload is characterized by increased catabolism. Easy and effective measures for reducing sodium load and fluid retention include choosing a hypotonic rather than isotonic maintenance fluid strategy (or avoiding these fluids when enough free water is provided through other sources) and dissolving as many medications as possible in glucose 5%.

## Introduction

Intravenous fluid therapy is associated with a broad spectrum of detrimental consequences. Well-known examples are the different clinical problems associated with the use of NaCl 0.9% and specific colloid solutions [1–4]. The best documented and most serious side effect of fluid therapy remains fluid overload, which is an independent risk factor for morbidity and mortality in critically ill and surgical patients [5, 6]. Excessive fluid volume has always been considered the root cause of iatrogenic fluid overload and has become a major research topic in the fields of perioperative and critical care medicine. However, another important and thus far largely neglected factor is the administered amount of sodium [7, 8]*.*

In the ICU, the largest burden of sodium is caused by maintenance fluid therapy prescribed to cover patients’ daily needs for fluids and electrolytes [9–11]. Furthermore, a substantial amount of sodium comes from fluid creep, the abundant fluids administered as a vehicle for intravenous medication or to keep intravenous lines open [9]. Since NaCl 0.9% is very often used for this purpose, high amounts of sodium can be administered inadvertently [9, 10, 12].

The aim of this narrative review is to draw attention to fluid retention caused by the large sodium amounts typically administered to hospitalized patients. We summarize the physiological background and explain why renal sodium handling is inefficient and energy-intensive. We attempt to quantify sodium-induced fluid retention and assess its clinical impact [7, 8]. Finally, we discuss various preventive and therapeutic options, paying specific attention to avoidable sodium sources.

## The hospital is a “sodium-rich environment”

The Intersalt study evaluated sodium intake in 52 populations throughout the world and found a wide range of sodium excretion (and thus intake) with medians from 1 to 246 mmol per day [13]. According to most health organizations, a healthy diet contains no more than 2.3 g (100 mmol) per day and provides an ample daily water intake of 2–2.5 L for the efficient excretion of solutes. As a result, the mean sodium-to-water ratio of a healthy diet is around 40–50 mmol per liter. When compared to normal dietary sodium intake, substantial amounts of sodium are being administered daily to hospitalized patients.

A significant source of sodium in the hospital is the extensive use of maintenance fluid therapy. A prospective single-day point prevalence survey, conducted in 46 Australian and New Zealand ICUs, demonstrated that maintenance and replacement fluids are responsible for 30.9% of the total daily sodium administration of 220 mmol (5 g) and—contrary to common belief—a much more significant source of sodium than resuscitation fluids [10]. We confirmed that maintenance and replacement fluids are the largest source of sodium administration in a retrospective study in 14,654 patients, showing that these fluids accounted for a larger fluid burden than resuscitation fluids, blood products and enteral nutrition together [9]. Sodium burdens caused by maintenance fluid therapy have increased substantially due to the ubiquitous practice of prescribing isotonic solutions for this purpose. Table 1 illustrates the large difference from the sodium content of a healthy diet [14]. For example, even the amount of sodium in 2 L of Ringer’s lactate exceeds the median daily sodium intake of the country with the highest sodium intake in the world.

**Table 1:** Sodium burdens of maintenance fluid regimens with different intravenous isotonic and hypotonic solutions

Intravenous solution (sodium concentration per liter)	Daily sodium intake if a typical amount of 2 L of this solution is used as maintenance fluid	
	Sodium (g/day)	Sodium (mmol/day)
1 L NaCl 0.9% (3.5 g/154 mmol)	7	308
1 L PlasmaLyte^®^ (3.2 g/140 mmol)	6.4	280
1 L Ringer’s lactate (3 g/130 mmol)	5.9	260
1 L NaCl 0.45% in G5% (1.8 g/77 mmol)	3.5	154
1 L Glucion 5%^®^ (1.2 g/54 mmol)	2.5	108
1 L Maintelyte^®^ (0.9 g/40 mmol)	1.8	80
**Clinical nutrition**		
Typical (par)enteral nutrition solution (± 1 g/40–50 mmol)	1.8–2.3	80–100

*G5*% glucose 5%

A second major—this time unintentional—source of sodium in the hospital is the custom of dissolving medications in NaCl 0.9% to enable intravenous administration. Bihari et al. demonstrated that drug boluses accounted for 12.3% of total sodium administration and drug infusions for 8.6% [15]. We previously reported that fluid creep, the cumulative unintentional fluid volume used as a vehicle for dissolving medication or to keep intravenous lines open was the largest source of daily fluid administration, representing 32.6% of total fluid intake [9]. Both studies unmasked fluid creep as the largest source of inadvertent sodium administration in the hospital. The need to dissolve intravenous medication comes in addition to the fact that many medications themselves contain sodium, mostly to make the active substance more soluble in water [16]. For example, valproate in its salt form is very soluble in water (1:0.4) compared to its minimal solubility (1:800) in acidic form. A comprehensive list summarizing the sodium content of most commonly used drugs in the ICU shows that antibiotics in particular contain large amounts of sodium [12]. Effervescent medicines are also well-known for their high sodium content [17].

## The physiological handling of an abrupt increase in sodium administration

### Kidneys excrete a sodium load inefficiently, which leads to fluid retention

As early as the nineteenth century, it was demonstrated that there is a delay of 3 days before the kidneys are able to excrete sodium at the rate of intake following abrupt sodium load [18, 19]. Later experiments determined that healthy kidneys of normal volunteers on a low-sodium diet (0.5 g, or ± 20 mmol per day) adapted surprisingly slowly when sodium intake was increased to about 3.2 g per day (± 150 mmol) [20]. Five days were needed to realign renal excretion with intake, with only about half of the excess sodium intake being excreted on the first day. The positive sodium balance caused fluid retention and an increase in body weight of over 1 kg. Once baseline dietary intakes were restored, it again took several days to lose the gained weight. This slow rate of renal sodium handling is remarkable considering that healthy kidneys filter the entire plasma volume six times per day. Yet, they seem unable to manage an additional sodium load of a few hundred millimoles above the usual dietary intake. From an evolutionary viewpoint, however, this makes perfect sense. Indeed, compared to the risks of dehydration and sodium loss, sudden (voluntary) peaks in sodium intake have been a virtually non-existent problem throughout evolution. It is thus understandable that herbivores and omnivores, including humans, favor the conservation of water and sodium at the expense of a poor ability to excrete sodium [21]. This is illustrated by the fact that the nephron lacks a dedicated mechanism for active sodium secretion, while the retention of sodium is the physiological centerpiece of aldosterone-mediated volume retention [22].

### Retaining water is an energy-demanding, catabolic process

After an episode of sodium gain, total body water osmolality needs to be restored by the accrual of free water. Counterintuitively, it is not water ingestion (thirst-induced or otherwise) that is the most important effector in realigning osmolality following increased salt intake [23, 24]. Human subjects were even shown to ingest less water after a long-term increase in sodium intake [23]. The main mechanism for generating an endogenous water surplus is the ability of the kidneys to reduce free water loss by concentrating urine [23, 24]. Unfortunately, the reabsorption of solute-free water in response to an increased sodium intake comes at a price. Several studies showed that urine concentration requires the accumulation of urea in the renal medullary interstitium in order to deliver the necessary osmotic driving force.[23, 24] The release of extra urea is an energy-intense process that requires a marked, glucocorticoid-driven catabolic reprioritization and a higher energy expenditure [23, 25]. In animal studies, this process was even shown to contribute to body weight loss [24].

### Excreting solutes is difficult if not enough free water is provided

Eventually, superfluous sodium will need to be excreted. In view of the evolutionary adaptations discussed above, the renal ability to concentrate sodium is limited to about two times the plasma sodium concentration. This is in sharp contrast to other solutes that can be concentrated 10 to 1000 times above their plasma value [21]. When the maximum level of renal sodium concentration is reached (250–300 mmol per liter), the kidneys require the ingestion of additional free water to increase urinary volume and excrete additional sodium [21]. Sodium excretion is thus more difficult when ample free water is unavailable [26]. Limited access to water is a typical condition of many surgical and critically ill patients. In recent decades, a marked shift in the incidence of ICU-acquired dysnatremias from hyponatremia to hypernatremia was observed in two large Dutch cohorts [29]. Responsible factors are suspected to include increasingly restrictive fluid policies, diuretic use and the use of sodium-rich infusions.

Moreover, apart from sodium, other solutes also need to be excreted. Many critically ill patients exert osmotic diuresis due to a disturbed glucose metabolism, osmotic therapies or increased urea levels caused by (hyper)alimentation and ongoing catabolism [27, 28]. The necessity of excreting solutes is the main reason why it is not desirable to omit hypotonic maintenance fluids when other sources of water are absent. Hypotonic solutions provide the free water necessary to excrete the sodium and other solute burdens that are common in hospitalized patients.

### It takes two to tango! What is the role of chloride?

An important aspect is that sodium is usually co-administered with chloride. It is well-recognized that sodium chloride—but not other sodium salts such as sodium bicarbonate—causes renal vasoconstriction, leading to a decrease in renal blood flow and glomerular filtration rate [30, 31]. This explains why, after a bolus of 2 L of Hartmann’s solution (Na 131 mmol/L, Cl 111 mmol/L), both water and sodium were excreted more efficiently than an equal amount of NaCl 0.9% [32]. The co-administration of sodium and chloride can be partially avoided by the use of balanced solutions (e.g., Ringer’s lactate, Hartmann’s solution), which could therefore be superior to NaCl 0.9% in terms of fluid retention. Balanced solutions typically contain 100–110 mmol of chloride per liter compared to 154 mmol in NaCl 0.9%. However, in the discussion on sodium overload due to maintenance fluid therapy, the value of balanced solutions should not be weighed against the use of NaCl 0.9%, but against the use of a hypotonic fluid strategy. Balanced isotonic solutions will still impose much higher sodium and chloride burdens than hypotonic fluids (see also Table 1) [9]. It is unlikely that the chloride content of even unbalanced hypotonic maintenance fluids (e.g., NaCl 0.18–0.45% in glucose or dextrose 5%) cause much fluid retention, since their chloride levels are well below that of human plasma and approximate dietary intake. Notably, the advantage of balanced solutions over NaCl 0.9% is further reduced when the necessary potassium administration is provided in the form of potassium chloride (although this issue could be resolved by administering potassium phosphate) [7, 9, 33].

## The clinical consequences of sodium administration

### Compared to sodium-poor alternatives, sodium-rich maintenance fluids lead to significant increases in fluid balance, independent of volume

Quantifying the effect size of sodium-induced fluid retention due to fluid therapy is challenging. Different experiments showed that NaCl 0.9% is excreted more slowly than more hypotonic solutions. However, these studies only assessed the short-term effect of a single-fluid bolus [32, 34]. In a study conducted over six consecutive days, 12 surgical patients received 3 L/day of either NaCl 0.9% or dextrose 5% [35]. The NaCl 0.9% group retained around 1000 mmol of sodium and developed a positive fluid balance exceeding 3 L as early as the second postoperative day. The dextrose arm experienced mild hyponatremia on the first postoperative day, which improved rapidly as negative fluid balances (approximately 2 L by day 4) developed. Unfortunately, the study design varied both fluid volume and sodium content, making it impossible to assess fluid accumulation by sodium administration separately.

**Fig. 1:**
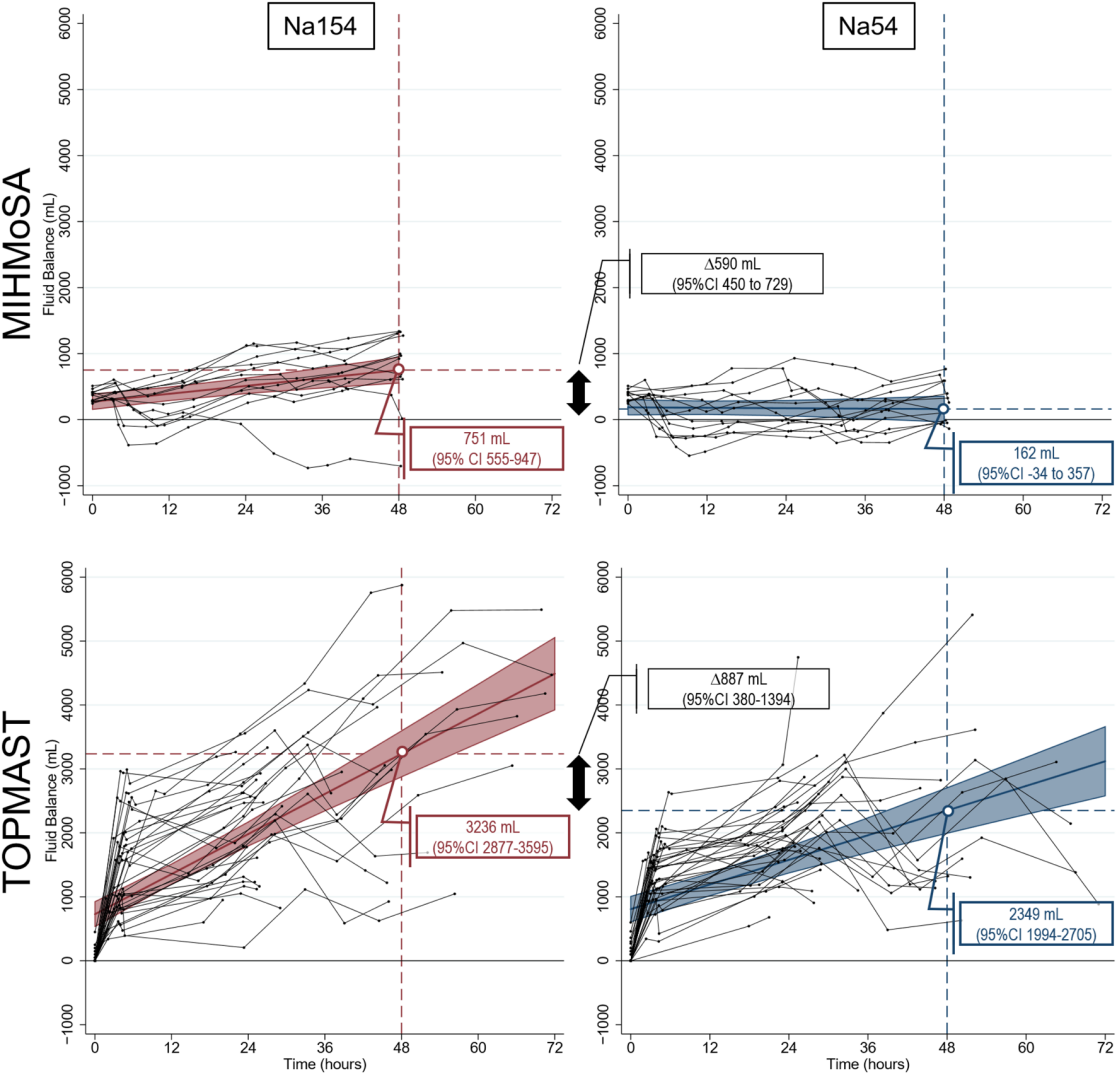
Cumulative fluid balance of the MIHMoSA and TOPMAST trials. Both in healthy volunteers (MIHMoSA) and in patients undergoing major surgery (TOPMAST), fluid retention was significantly higher in the treatment arm receiving maintenance fluids containing 154 mmol/L of sodium (compared to 54 mmol/L). Compared to healthy subjects, the patient cohort had a more positive fluid balance, no matter the study fluid, partly due to other net fluid input (± 1.5 L) and partly due to the physiological response to hypovolemia, capillary leakage, etc. For details: see text. Adapted from Van Regenmortel et al. and Van Regenmortel et al., with permission [7, 8]

Two of our own studies, the MIHMoSA and the TOPMAST studies, had the primary goal of measuring the effect size of sodium-induced fluid retention using two common maintenance fluid regimens. During the MIHMoSA crossover experiment, cumulative fluid balance was assessed in 12 healthy volunteers who refrained from any oral intake during two separate study periods of 48 h. They were administered maintenance fluids with a sodium concentration of 154 (Na154) or 54 mmol/L (Na54), administered at 25 mL/kg/day [8]. Cumulative fluid balance after 48 h was 590 mL (95% CI 450–729) more positive in patients receiving Na154. Using an almost identical study design, the same fluids and similar infusion rates, the double-blind randomized controlled TOPMAST trial evaluated cumulative fluid balance in 70 patients with normal kidney function who had undergone major thoracic surgery [7]. The estimated cumulative fluid balance at 48 h was 887 mL (95% CI 380–1394) more positive in the Na154 arm, despite almost identical non-study fluid sources and fluid losses through drain outputs. As a result, the fluids’ sodium content was considered responsible for an additional fluid retention of 22% (95% CI 10–34%) of its infused volume. Figure 1 shows the cumulative fluid balance encountered in the two studies as reported in the original papers, rescaled for optimal comparison.

### Is sodium-induced fluid retention associated with poorer clinical outcomes?

A positive cumulative fluid balance has long been associated with undesirable outcomes in septic and critically ill patients [5]. In the perioperative setting, restrictive fluid regimens were convincingly shown to be superior to more liberal fluid policies [36, 37]. The specific clinical impact of sodium-induced rather than volume-induced fluid retention is difficult to demonstrate in view of the scarce dedicated research. Yet, various pieces of evidence hint at its clinical importance. First, a body weight gain of 2 to 3 kg due to sodium-rich perioperative fluid therapy compared to sodium-poor alternatives was shown to be associated with increased perioperative morbidity [38]. Second, a prospective observational study in 50 mechanically ventilated patients showed that a positive sodium balance was associated with a next-day reduction in the PaO_2_/FiO_2_ and increased duration of mechanical ventilation [15]. Interestingly, these adverse respiratory outcomes were not related to the cumulative positive fluid balance. Finally, in TOPMAST, treatment with Na154 was halted in 17% of cases due to clinical or radiographic fluid overload for which diuretics were needed, compared to 3% in the Na54 group (*p* = 0.05) [7]. Even less research has addressed the clinical impact of the catabolic generation of urea to improve urine concentration and the importance of providing enough free water to assist in sodium excretion. In view of the extensive use of sodium-rich solutions in the hospital, appropriate scientific efforts are urgently needed.

### Fluid balance needs a balanced view

It cannot be ignored that a positive fluid balance is often a marker of illness severity rather than the cause of harm itself. Indeed, even a markedly positive fluid balance is frequently unavoidable, as it is not merely the result of imprudent fluid therapy, but also a combination of physiological processes responding to certain clinical realities such as hypovolemia, vasodilation, and capillary leakage. As such, a patient’s fluid balance can be positive in the presence of euvolemia, or even intravascular hypovolemia. Figure 1 shows much higher cumulative fluid balances in both TOPMAST treatment arms compared to the healthy volunteers in the MIHMoSA experiment. This difference could not be explained by off-study net fluid input. It seems that, in the TOPMAST study, intravenous fluids were retained by the kidneys to counterbalance vasodilation, hypovolemia and/or the increased endothelial permeability associated with surgery/anesthesia. Neglecting this reality and striving for an absolute “magic number” or even a zero fluid balance could be harmful. The results of the RELIEF trial, in which patients in the restrictive fluid arm were more prone to develop AKI, remind us that the pendulum of fluid restriction can easily swing too far [39]. Despite the need for a cautious and nuanced consideration of cumulative fluid balance, the fact remains that iatrogenic fluid overload certainly exists and—in the absence of a more optimal parameter—many clinicians will use it to judge the need for clinical intervention. Therefore, we are convinced it is important to avoid any additional factors that have the potential to further derail fluid balance. Sodium-induced fluid retention is one of those factors.

## Streamlining the sodium pandemonium

Preventing fluid overload is notoriously difficult, and determining the optimal amount of fluid is one of the most challenging clinical decisions in critically ill and surgical patients. In contrast, avoiding unnecessary sodium overload might be the low-hanging fruit. Reducing the sodium burden could thus be a straightforward and feasible option in the battle against iatrogenic fluid overload. Fortunately, the search for avoidable sources of sodium is not extremely challenging, as two of them are hidden in plain sight.

### Step 1: cut the creep!

Dissolving as many medications as possible in glucose or dextrose 5% can dramatically reduce unintentional sodium administration. Oral intake or enteral administration could be valuable alternatives to drugs with high oral bioavailabilities. Table 2 provides the results of a medication-per-medication literature search of the most common medicines used in critical care, whether intermittently or continuously through syringe pumps. Notably, the use of NaCl 0.9% as a solvent is mandatory for surprisingly few medications. For the majority, it is simply an unfortunate habit. A prospective before-and-after study in a tertiary ICU demonstrated the feasibility of decreasing the total sodium (and chloride) burdens by intervening in fluid creep and in the type of maintenance fluids [40]. The amount of sodium administered daily decreased by almost half to a median of 109 mmol (IQR 77–288), leading to a decrease in daily fluid balance and electrolyte disturbances.

**Table 2:** Most used medication in the intensive care unit to be diluted for continuous infusion, slow bolus administration or both. The stability of the diluted product is dependent on its end concentration and environmental temperature. The product’s stability in its compatible diluent has been confirmed for the conventional administration times and volumes. For detailed data, we refer to each drug’s SmPC (summary of product characteristics) or relevant databases

	NaCl 0.9% mandatory	G5% or D5% or NaCl 0.9% equally possible	G5% or D5% mandatory	Administration in its pure form using a syringe pump feasible
Anti-infectives				
Continuous or prolonged (4-8 h) infusion		Benzylpenicillin		
		Cefepime dihydrochloride		
		Ceftazidime pentahydrate		
		Meropenem trihydrate^a^		
		Piperacillin–tazobactam		
		Temocillin		
		Vancomycin^b^		
Short infusion (15–90 min, depending on product)	Amoxicillin–clavulanate^c^	Benzylpenicillin		
	Acyclovir^d^	Cefepime dihydrochloride		
		Ceftazidime pentahydrate		
		Ciprofloxacin^e^		
		Flucloxacillin		
		Meropenem trihydrate^a^		
		Piperacillin–tazobactam		
		Sulfamethoxazole–trimethoprim^f^		
		Temocillin		
		Vancomycin^b^		
Vasoactive and antiarrhythmic medication				
Continuous infusion	Somatostatin	Adrenaline tartrate or HCl^g^	Amiodarone HCl	Isosorbide dinitrate
		Dobutamine HCl	Norepinephrine^k^	Labetalol
		Isosorbide dinitrate^h^		Nicardipine
		Labetalol		Nimodipine
		Milrinone		Urapidil HCl
		Molsidomine		
		Nicardipine^h^		
		Urapidil HCl		
		Clonidine HCl		
Short infusion (15–90 min, depending on product)		Labetalol	Amiodarone HCl	
Sedatives, analgetics and antiepileptic agents				
Continuous infusion		Alfentanyl		Alfentanyl
		Clonidine HCl		Fentanyl citrate
		Dexmedetomidine HCl		Ketamine
		Fentanyl citrate		Midazolam HCl
		Ketamine		Morphine HCl
		Midazolam HCl		Sufentanyl citrate
		Morphine HCl		
		Remifentanyl		
		Sufentanyl citrate		
		Thiopental sodium		
		Valproate sodium		
Short infusion (15–90 min, depending on product)	Phenytoin sodium	Levetiracetam		
		Valproate sodium		

	NaCl 0.9% mandatory	G5% or D5% or NaCl 0.9% equally possible	G5% or D5% mandatory	Administration in its pure form using a syringe pump feasible
Other common medications used in the ICU				
Continuous infusion		Cisatracurium besylate		Bumetanide
		Concentrated electrolytes (KCl, MgSO4)		Cisatracurium besylate
		Furosemide^i^		Concentrated electrolytes^j^
		Heparin sodium		Furosemide
		Methylprednisolone sodium succinate		Heparin sodium
		*N*-Acetylcysteine		
		Regular insulin		
Short infusion (15–90 min, depending on product)		Bumetanide		
		Concentrated electrolytes (KCl, MgSO4)		
		Furosemide^i^		
		*N*-Acetylcysteine		
		Methylprednisolone sodium succinate		

*D5*% dextrose 5%

*G5*% glucose 5%

^a^Some sources report a stability of less than 4 h in G5%

^b^After reconstitution with aqua

^c^After reconstitution with aqua or NaCl 0.9%

^d^Glucose 5% possible but probably less stable because of Maillard reaction

^e^Mostly commercially prediluted, solvent differs by brand

^f^Minimal necessary volume for dilution: 400 mg + 80 mg/5 mL in 75 mL

^g^pH of G5% needs to be around 5.5, otherwise reduced stability

^h^Avoid polyvinylchloride (PVC) when diluted in NaCl (sorption to PVC 9 pH of G5% needs to be around)

^i^Precipitation possible if pH < 5.5–7

^j^Depending on the initial concentration

^k^Although strictly not impossible to dissolve in NaCl 0.9%, G5% is recommended for protection against significant drug loss due to oxidation

### Step 2: adopt a hypotonic maintenance fluid strategy without losing sight of hyponatremia

First, we want to emphasize that the necessary amounts of water and electrolytes should ideally be administered orally or enterally. Intravenous maintenance fluid therapy should only be prescribed in situations where patients are able neither to ingest food or fluids nor to receive (par)enteral nutrition. As soon as other sources provide enough free water to excrete sodium and other solutes, maintenance fluids become redundant. On the other hand, banning all maintenance fluids from the hospital or the ICU will lead to many patients being unable to excrete the necessary solutes, especially because of the displacement of intravascular fluid towards the interstitial space. The ensuing risk of acute kidney injury, encountered after a too-restrictive fluid policy, is unacceptable [39]. We want to stress the fact that maintenance fluids are not the same as resuscitation fluids (isotonic by nature, and frequently guided by hemodynamic monitoring) or replacement fluids, which are prescribed to cover lost fluids or ongoing fluid losses, for example in the event of diarrhea, fever, dehydration, losses through different types of surgical drains, and so on [41, 42]. Maintenance fluids should be prescribed only to cover daily needs for water and electrolytes and should therefore be as close as possible to a healthy dietary intake (Table 1). From that point of view, they should contain free water and their sodium content should be low.

The other side of the coin and the most feared trade-off of a hypotonic maintenance fluid strategy is the occurrence of hyponatremia. This is the reason why maintenance fluids’ ideal sodium content remains a matter of heated debate [43–47]. In-hospital hyponatremia mostly develops due to the actions of antidiuretic hormone (ADH), an osmoregulatory hormone that protects against hyperosmolality. In hypovolemia (occult or otherwise), however, volume regulation is physiologically prioritized over osmoregulation. When ADH is subsequently upregulated in the absence of an osmotic stimulus and the hypovolemia is not treated adequately with replacement or resuscitation fluids, hyponatremia can occur [43]. Pediatric patients are particularly sensitive to the development of symptoms (mainly neurological) caused by this electrolyte disorder. The question remains whether it is justified to advocate the use of isotonic maintenance solutions for all hospitalized patients to avoid hyponatremia in a minority of them. This is especially questionable in the critical care setting, where other sodium sources are present and sodium is measured multiple times daily through point-of-care analysis [43, 45, 48, 49]. Currently, there is little proof of clinically relevant maintenance fluid-induced hyponatremia. Even in the pivotal pediatric trial on the subject, no patients developed symptomatic hyponatremia [50]. Seizures were encountered in 7/338 patients (2%) in the hypotonic arm, compared to 1/338 patients (0.3%) in the isotonic arm (*p* = 0.07), but all instances occurred in patients with known seizure disorders. In the adult patients of the TOPMAST trial, most instances of hyponatremia were mild and none was symptomatic. Bihari et al. showed that a reduction of sodium and chloride in maintenance fluids and fluid creep led to no differences in either the rate or severity of hyponatremia [40].

**Fig. 2:**
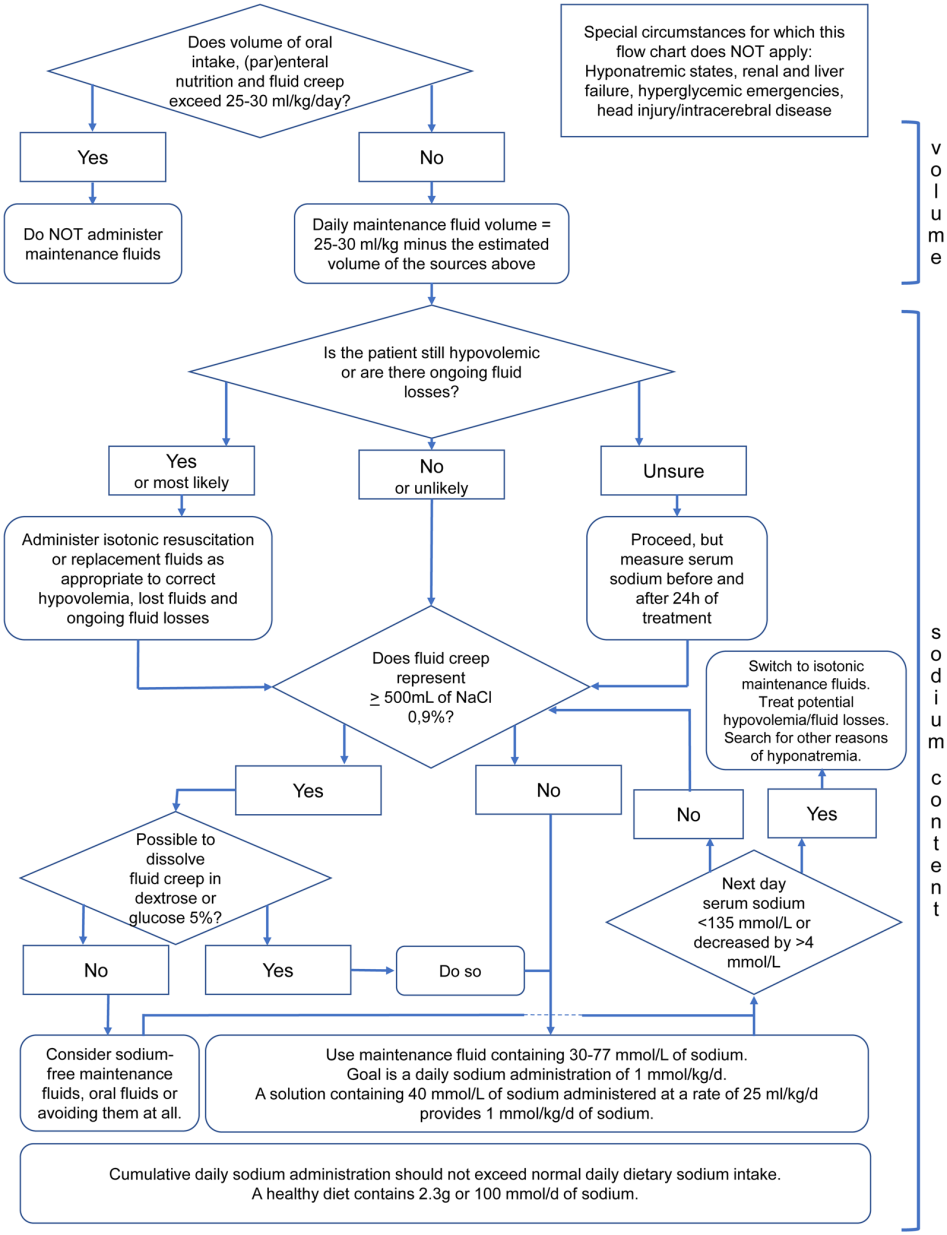
Suggested maintenance fluid strategy for in-hospital patients, especially those who are at risk of fluid overload or hyponatremia

In order to take all the elements above into account when prescribing maintenance fluid therapy, we propose the algorithm in Fig. 2. The starting point is the guideline-recommended dose of 1 mmol of sodium per kg of body weight and 25 mL per kg of fluid volume per day [42, 51]. Our proposed flowchart accounts for the presence of other fluid sources, for sodium burdens caused by fluid creep and for the risk of hyponatremia caused by suspected or confirmed hypovolemia. The algorithm takes preventive action towards hyponatremia by including a vigilant attitude towards hypovolemia (occult or otherwise) and ongoing fluid losses and more proactive treatment of at-risk patients. Fluid-depleted patients need isotonic resuscitation or replacement fluids before hypotonic maintenance fluids are considered. When fluid status is difficult to assess, we recommend measuring serum sodium at least before the start and after 24 h of hypotonic maintenance treatment. When hyponatremia ensues, a switch to isotonic maintenance fluids is reasonable.

We again point out that popular balanced solutions such as Ringer’s lactate are efficient in preventing hyperchloremia but have little added value over NaCl 0.9% in terms of sodium content (Table 1). On the other hand, intravenous solutions with low osmolality cause venoirritation, which is the reason that hypotonic fluids (NaCl 0.45% or lower) are almost without exception dissolved in glucose or dextrose. For example, the addition of glucose 5% increases the osmolality of NaCl 0.45% from a poorly tolerated 154 mOsm/kg to 432 mOsm/kg (without changing the tonicity of the fluid). In the absence of other caloric intake, this has the additional advantage of providing a basic level of caloric intake and thereby preventing starvation ketosis. Meanwhile, hyperglycemia is a well-known risk factor for morbidity in critically ill patients and should be prevented by careful glycemic control, especially in critically ill patients [52, 53].

### Step 3: therapeutic options and the challenge of removing sodium without water

On a therapeutic level, it is difficult to remove sodium once administered. This is especially true as many diuretics result in diuresis in excess of natriuresis, causing hypernatremia [54]. Just like hyponatremia, hypernatremia is a common problem in the hospital and was found to be an independent predictor of increased mortality [29, 55, 56]. To avoid losing more water than sodium when using loop diuretics, the addition of indapamide has been successfully proposed [57]. Indapamide is a thiazide-like diuretic that acts on the Na–Cl symporter in the distal convoluted tubule. A prospective single-center study in 40 fluid-overloaded patients in the ICU demonstrated that the co-administration of 1 mg/kg of furosemide and 5 mg of enteral indapamide led to greater natriuresis compared to treatment with furosemide in monotherapy [57]. Although this concept remains untested in a trial with true clinical endpoints, it seems a feasible therapeutic option. Since thiazide diuretics are associated with an increased incidence of hyponatremia in the treatment of heart failure and lead to greater kaliuresis, these issues should be anticipated and treated appropriately [58].

## Conclusions

Although well-known in experimental research and in the field of hypertension, the dangers of unnecessary or unintentional sodium intake have not yet entered the clinical arena. In the light of the abovementioned findings, this seems unjustified. Evidence supports the hypothesis that high sodium burdens are a separate risk factor for fluid overload and induce a catabolic hormone profile. We therefore call for increased attention to be paid to sodium overload, especially since many of the preventive measures are easy to implement in clinical practice. Ideal starting points include adopting a maintenance fluid strategy that is low in sodium and provides enough free water, and avoiding NaCl 0.9% as the diluent for medication. Caution remains warranted with regard to the development of hyponatremia and hypovolemia. Now that the awareness of iatrogenic volume overload is being incorporated in daily practice and the measures for avoiding it are being pushed to their limits, we are convinced that the battle against sodium-induced fluid overload is the logical next step.

### Take home messages

Unphysiological amounts of sodium, surpassing normal dietary intake, are commonly administered to hospitalized patients through ill-considered maintenance fluid therapy and fluid creep. The notoriously difficult renal handling of this sodium overload leads to potentially harmful fluid retention. We call for increased attention to be paid to this this avoidable problem and suggest the use of low-sodium maintenance fluids (or their avoidance whenever possible) and recommend “cutting the creep” by avoiding NaCl 0.9% as the diluent for medication.
